# Propagule Pressure and Climate Contribute to the Displacement of *Linepithema humile* by *Pachycondyla chinensis*

**DOI:** 10.1371/journal.pone.0056281

**Published:** 2013-02-08

**Authors:** Eleanor Spicer Rice, Jules Silverman

**Affiliations:** Department of Entomology, North Carolina State University, Raleigh, North Carolina, United States of America; Stanford University, United States of America

## Abstract

Identifying mechanisms governing the establishment and spread of invasive species is a fundamental challenge in invasion biology. Because species invasions are frequently observed only after the species presents an environmental threat, research identifying the contributing agents to dispersal and subsequent spread are confined to retrograde observations. Here, we use a combination of seasonal surveys and experimental approaches to test the relative importance of behavioral and abiotic factors in determining the local co-occurrence of two invasive ant species, the established Argentine ant (*Linepithema humile* Mayr) and the newly invasive Asian needle ant (*Pachycondyla chinensis* Emery). We show that the broader climatic envelope of *P. chinensis* enables it to establish earlier in the year than *L. humile*. We also demonstrate that increased *P. chinensis* propagule pressure during periods of *L. humile* scarcity contributes to successful *P. chinensis* early season establishment. Furthermore, we show that, although *L. humile* is the numerically superior and behaviorally dominant species at baits, *P. chinensis* is currently displacing *L. humile* across the invaded landscape. By identifying the features promoting the displacement of one invasive ant by another we can better understand both early determinants in the invasion process and factors limiting colony expansion and survival.

## Introduction

A central challenge in invasion biology is identifying the mechanisms governing the establishment and spread of new invasive species. Where some invasive species remain innocuous or undetected for the duration of their establishment, others experience population increases and range expansion with adverse consequences to native taxa [1]. Because invasive species are often not studied until they negatively impact the environment, pinpointing the factors contributing to initial dispersal and subsequent spread is often retrospective and can prove challenging [2]–[4]. Furthermore, there are disparities in invasion ecology research, where short-term experiments are often terminal and long-term projects often take the form of inferences between past and present data without analytical studies throughout the actual invasion process [3], [5], [6].

While biotic resistance [7], [8], behavioral dominance [9], and propagule pressure (number of independent introductions and number of individuals introduced [10]; can be determinants of successful establishment, abiotic factors such as environmental suitability [11] can be predictors of persistence and spread [12]–[14]. Disentangling the factors governing the establishment and spread of invasive species is crucial to identifying and preventing the environmental impacts caused by these organisms [15], [16]. Ants make an excellent model for investigating these factors because they are a diverse family with a plethora of evolutionary histories and competitive tactics [17].

As with other invasive taxa [18]–[21], many ant species negatively impact native-species richness and diversity once established [22]–[24]. However, some native taxa manage to persist in invaded habitats, and different invasive species co-occur within the same habitat each capitalizing on different microclimates [25], [26], refugia [27], and nutrients [28], [29]. Furthermore, more than one invasive ant species can exploit the same disturbed habitat [30]. While the negative impacts of invasive ants are well documented, examples of native species recovering from or being unaffected by ant invasions are rare [31]–[33].

One invasive ant species with profound deleterious ecological effects within its introduced range is the Argentine ant (*Linepithema humile*). Spanning disturbed landscapes in six invaded continents, Argentine ants are on ISSG's “100 World's Worst Invasive Alien Species” list. Once established, Argentine ants are persistent and form large aggressive colonies, fostering hemipterans [34] and generally displacing native ants [35], [36], with cascading effects across many trophic levels [2], [37], [38].

A relatively new invasive ant, the Asian Needle Ant (*Pachycondyla chinensis*), is primarily restricted to the Eastern United States. It forms comparatively small colonies within both disturbed urban and undisturbed natural habitats and like the Argentine ant, *P. chinensis* has impermanent nests [39]. Its introduction into a novel area often coincides with declines in native ant fauna [39], including keystone seed dispersing mutualists, thereby reducing some local myrmecochorous plant abundance by 50% [40]. The mechanism by which *P. chinensis* displaces native ants is not known. *Pachycondyla chinensis* possesses a venomous sting causing human anaphylaxis, thereby posing an immediate human health threat as it spreads into anthropogenic habitats [41]. There have been no efforts to eradicate this ant, although applications of toxic baits appear to be a promising control methodology [42].

Our lab has studied *L. humile* within an urban office park for over a decade [43]. Beginning in 2008, we observed small numbers of *P. chinensis* in locations where other ant species were absent. This incipient *P. chinensis* population offered a rare opportunity to follow its potential spread and interaction with an established *L. humile* population. There is a strong seasonal effect on the distribution and range expansion of *L. humile* with occasional rapid establishment of native ant species when populations of *L. humile* are small [44]–[47], yet little is known of the impact of a recent invasive ant introduction on an established invader [48]. *Linepithema humile* relies on high worker numbers and highly aggressive behavior to establish and maintain territories [23], [49].

Across taxa, matching climatic conditions between native and invaded ranges is an important predictor for invasion success [50]–[54]. Climate is correlated with Argentine ant range expansion [55], [56] with habitats of introduction having similar climate to those within the native range (subtropical South America; [57]). Our field site in Raleigh NC is at the northern edge in the eastern US of the Argentine ant's climatic envelope, such that worker population sizes shrink during relatively cold winter months and resurge in late spring and summer [44]. *Pachycondyla chinensis*, in contrast, is native to regions in Asia with a temperate climate [58].

Another predictor of invasive species establishment is propagule pressure, whereby both the number of introductions and number of individuals introduced are correlated with invasion success [7], [59], [60]. In ants, propagule pressure contributes to establishment success [61]–[63]. Also, numerical and behavioral dominance facilitate the spread of *L. humile* [64]. Many studies employ historical data [65], [66], modeling [67], [68], or manipulative experiments [69]–[71] to observe an invasion path over time. By surveying the distribution of *P. chinensis* and *L. humile* over four years, we have a unique opportunity to track *P. chinensis* as it establishes and expands its range.

Here, we use a combination of field surveys and experimental approaches to test the relative importance of behavioral and abiotic factors in the establishment and spread of *P. chinensis* within habitat occupied by *L. humile*. We make the following predictions: 1) *P. chinensis* establish in territory occupied by *L. humile* during seasons of low *L. humile* abundance and activity, 2) increased *P. chinensis* propagule pressure during periods when *L. humile* is relatively inactive improves establishment success, 3) *L. humile* dominate food resources in areas of species overlap and 4) *P. chinensis* is more cold-tolerant than *L. humile*. We show that while *L. humile* is behaviorally and numerically dominant, *P. chinensis* is displacing *L. humile* by establishing nests during seasons when *L. humile* populations are low.

## Materials and Methods

### Study area and insect maintenance

We conducted our study on the grounds of a privately owned 47.37- hectare office park in Morrisville, North Carolina USA (35°51′11.37″N 78°49′36.74″W). No specific permits were required for the described field studies. Permission was granted by Duke Realty Co. Morrisville, NC. Argentine ants have infested this location for over a decade [43]. The climate is temperate (mean annual minimum, 1°C; mean annual maximum, 32°C) and is at the northern edge of *L. humile*'s invaded range in the eastern US [44], but is currently near the center of *P. chinensis*'s invaded range [41].Our lab has routinely sampled this location for the past thirteen years and only noted *P. chinensis* in 2008. In March–June 2008, we constructed a grid overlay across an aerial map of the park and used a random number generator to select 132 locations across the grid for pitfall sampling. Once each month for three months, we placed pitfalls in each location for 72 hours, and recorded ant species captured. For every location in which *P. chinensis* was captured, we performed extensive visual surveys working in a 20 m circle outward from the pitfall trap, inspecting all suitable nest sites and across ground cover for ants. This examination revealed *P. chinensis* nesting in close proximity (<1–5 m) to *L. humile* nests, which are frequently in pine needle mulch around the bases of willow oak trees (*Quercus phellos*) throughout the park. While *L. humile* is evenly dispersed across the ca. 35 hectares of developed land on the site, *P. chinensis* occurs patchily, initially occupying three approximately 10 m^2^ areas, 1.7 km apart on the developed land and four 5 m^2^ areas across surrounding mixed pine-hardwood forest. All ants collected for laboratory studies were held in plastic tubs coated with Fluon™ to prevent escape. Ants were provided artificial nests (95×15 mm Petri dish with moistened plaster base and covered with a tile) and a diet of 20% sucrose solution and freshly killed German cockroaches (*Blattella germanica*). We maintained all ants at 26°C±1°C, 50%±5% RH, and a 12∶12 L∶D.

### Evidence of *L. humile* and *P. chinensis* across seasons

We conducted monthly ant surveys from March 2009 to June 2011 to identify any differences in seasonal occurrence between *P. chinensis* and *L. humile*. We sampled within the mulch surrounding trees where nests of both ants occurred. Based on the initial visual and pitfall surveys described above, two locations (∼1 km apart) with a total of 22 sites having both *P. chinensis* and *L. humile* were designated “species overlap” sites and two groups with only *L. humile* evident (n = 22) were designated “*L. humile*-only” sites. Surveys consisted of removing the pine mulch from around each tree base in a circle with a 90 cm radius, beginning at the point of contact between tree and ground. We removed mulch in a width of 10 cm at a time, and each 10×90 cm section was searched for ants. We replaced the mulch after each survey. *Linepithema humile* can amass very high worker densities in the summer months, while *P. chinensis* colonies remain comparatively small. To avoid destructive sampling—and because we were measuring ant presence and not ant abundance—we chose “cluster” as our unit of measure to account for each species' disproportionate worker number. A cluster consisted of a discrete group of four or more *P. chinensis* workers or a discrete group of *L. humile* workers surrounding a queen. We recorded the number of clusters per tree base. We measured tree circumference for all trees and found no differences across treatments (mean 137.82±4.15 cm, t = 1.81, p = 0.27). We expected that clusters of both ant species would be present in the spring months beginning in March. We conducted a multivariate test (PROC GLM) on the difference between the mean number of ant clusters within consecutive months for each species from March–July ('09), February–July ('10), February–June ('11) to determine if one species appeared earlier in the year than the other. Each species was compared alone, across the three years (SAS v.9.8, SAS Institute 2009).

We also conducted annual visual presence/absence surveys to determine whether *P. chinensis* displaced *L. humile*. We pulled back the pine needle mulch 90 cm from the base of each tree (n = 100) along the invaded area in the office park. We searched the ground for ants in the same manner as above. While we recorded ant clusters in the seasonal surveys, we recorded worker presence (at least one worker) in this annual survey. In early June (a time of activity for both species) of each year, we recorded *L. humile-*only presence (given a score of 3), species overlap (score = 2), or *P. chinensis*-only presence (score = 1) at the base of each tree. One tree was devoid of ants for the duration of the study and so was eliminated from the analysis. Controlling for year, we then ran a Cochran-Mantel-Haenszel correlation test between year and score (PROC FREQ) (SAS v.9.8, SAS Institute 2009).

### Behavior and competition at baits

We measured bait discovery and domination along six 60 m transects occupied by either or both *P. chinensis* and *L. humile*. We placed bait cards (7.6×12.7 cm) every 5 m along each transect for a total of 12 bait cards per transect, two transects per site designation (*P. chinensis* only, *L. humile*-only, or area of species overlap). Each bait card received 0.5 g bait. Bait consisted of a mixture of equal parts tuna (in oil) and honey. We recorded the number of *P. chinensis* and *L. humile* at each bait card every 15 minutes for 3 hours. We sampled between June and September, during the season of peak activity for both species.

We recorded aggressive behaviors if more than one species was on a bait card. Aggressive behaviors included open mandibles, biting, gaster flexing, and chasing. If one species discovered a bait card but was replaced by another species for two or more observation periods, we recorded that as a “displacement event.” Other ant genera (*Formica sp.*, *Solenopsis invicta*, *Brachymyrmex sp.*, and *Prenolepis imparis*) occur at our field site and thus bait dominance through displacement could occur in areas of non- *P. chinensis*-*L. humile* overlap. Prior to sampling, we conducted extensive visual and bait surveys of the area around each transect to classify each location as “*P. chinensis* only,” “*L. humile* only,” or *P. chinensis/L. humile* “species overlap.” These classifications were used for data analysis with a total of 18 *P. chinensis*-only baits, 13 *L. humile*-only baits, and 17 baits occupied by both *P. chinensis* and *L. humile*.

We recorded the time to bait discovery by each species and compared this across sites occupied by one or both species. We compared mean time to discovery across each species for all overlap status groups using Analysis of Variance with Tukey's HSD means comparison. We recorded the proportion of baits (n/total) discovered and dominated by each species at *P. chinensis*-only, *L. humile*-only, and *P. chinensis*/*L. humile* overlapping sites. We analyzed data for baits that were discovered and baits that were dominated separately. We arcsine-square root transformed data before performing an Analysis of Variance and compared treatment means using Tukey-Kramer's pairwise means separation.

### Effect of colony size and order of establishment on ant species displacement and worker survival

**Figure 1:**
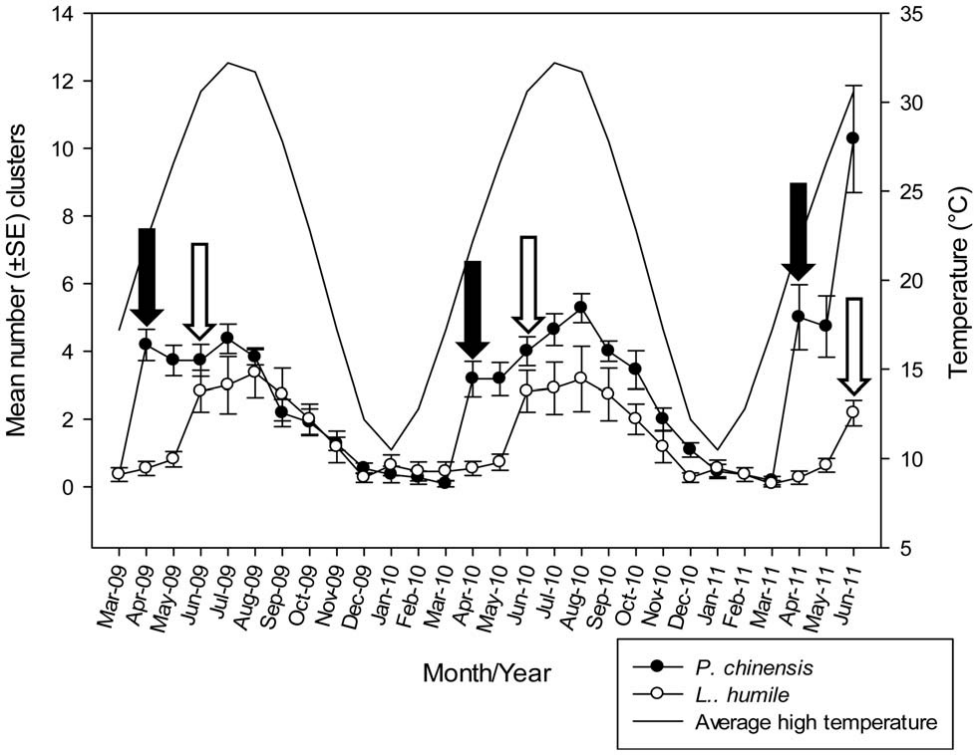
Seasonal abundance of *P. chinensis* and *L. humile*, with mean (±SE) clusters of ants at sampling sites each month and average maximum monthly temperature from March, 2009 to June, 2011. Black arrows indicate significant (PROC GLM p<0.001) numbers of *P. chinensis* (compared to previous month) and white arrows indicate significant (PROC GLM p<0.001) numbers of *L. humile* compared to that of prior month.

We recorded the outcomes of interactions between *L. humile* and *P. chinensis* at different colony ratios when each species was either an intruder or resident. We established *L. humile* colony fragments of 25 (n = 10), 50 (n = 10), and 125 (n = 10) workers, each with one queen. We also established colony fragments of 25 *P. chinensis* workers plus one queen (n = 30). We placed ants in a Fluon-lined plastic container (15×15×4 cm) furnished with a 95×15 mm Petri dish nest filled with moistened shredded coconut husk substrate, water, 25% sucrose solution, and freshly-killed German cockroaches maintained at 26°C±1°C, 50%±5% RH, and a 12∶12 L∶D. This temperature is the approximate average temperature for the month of *L. humile* activity onset in Morrisville, NC (see Fig. 1), to mimic field conditions during nest founding. Ants acclimated to test containers for four days, after which we recorded living workers and their location within the nest (presumably within optimal habitat). We recorded ants within, on top of, or under the Petri dish, within the water tube, or on the sucrose tube. We then added heterospecific ants to each of these containers; 25 *P. chinensis* plus one queen to the different density *L. humile* treatments and 25, 50, or 125 *L. humile* plus one queen to the containers with resident *P. chinensis* (n = 10 per ratio and primary establishment treatment). We aspirated intruders into a tube and gently tapped them into each container. We also performed single species controls (n = 12).

Seven days later, we recorded the number of living and dead workers and queens and the location (nest) where living ants of each species occurred. We arcsine-square root transformed the proportion of surviving individuals of each species within each replicate before performing Analysis of Variance and compared treatment and control means using Tukey-Kramer's pairwise means separation test. We recorded nest displacement as 0 or 1 (0 = individuals remained in established nest, 1 = individuals elsewhere). We arcsine-square root transformed nest displacement score before performing ANOVA and compared treatment means with Tukey-Kramer's pairwise means separation test.

### Reappearance of *L. humile* following removal of *P. chinensis* from the field

We considered that the disappearance of *L. humile* from locations within our field site might be a consequence of an altered habitat (e.g., reduced food, poor nesting substrate) rather than displacement by *P. chinensis*. Therefore, we provided a direct measure of the impact of *P. chinensis* on *L. humile* by removing *P. chinensis* from plots and recording the return of any *L. humile*. We selected five plots in locations that for the past three consecutive years were solely occupied by *P. chinensis*. *Linepithema humile* were recorded in these plots prior to the three years of *P. chinensis* occupancy and persisted on the periphery of these plots, with nests occurring between 1 and 10 meters away from each *P. chinensis* plot. Prior sampling revealed every tree base nest site was occupied by either species across the habitat. We first sampled *P. chinensis* and *L. humile* along a transect in each plot every 3 m using bait cards (7.6×12.7 cm) with 0.5 g tuna in oil (n = 12). We recorded ant species presence (0 = absent, 1 = present) 30 minutes later. We then killed *P. chinensis* in three plots with the granular insecticidal bait Maxforce® Complete (AI = hydramethylnon, 1.00%) dispensed at a label rate of 28.35 g per 4.6 m^2^. Maxforce® Complete granule size is 1–2 mm and can be retrieved by *P. chinensis*, but not *L. humile*, thus largely precluding *L. humile* from the negative impacts of the bait [72]. We included two *P. chinensis-*infested plots as untreated controls (n = 16 bait cards). We then re-sampled all plots 1, 3 and 14 days later and analyzed data with a Cochran-Mantel-Haenszel chi-square correlation test controlling for treatment and site (PROC FREQ). We calculated Agresti-Coull confidence intervals for the proportion of baits with *L. humile* present.

### Cold tolerance of *L. humile* and *P. chinensis*

We hypothesized that *P. chinensis* appear earlier in the season than *L. humile* because they are more cold-tolerant. We conducted a laboratory experiment to determine the survival of *L. humile* and *P. chinensis* at low temperatures. We placed 50 workers, one queen, and some brood of *L. humile* and 50 workers and some brood of *P. chinensis* into separate Fluon-lined plastic containers (12.5×12.5×5 cm). Because *P. chinensis* queens were found in few field clusters, we omitted queens from *P. chinensis* treatments. *Linepithema humile* queens cannot produce a functional nest without workers [73], so complete worker mortality in our experiment would result in the demise of the nest. Each container had a fine mesh lid and bottom to promote air flow. We provided ants with a moistened plaster nest (95×15 mm) covered with a tile, water in a small culture tube blocked with a moist cotton plug, 20% sucrose in a small culture tube blocked with a cotton plug, and freshly killed German cockroaches. We replaced water, sucrose, and cockroaches each week at the time of data collection.

Ants acclimated to their new environment at 26°C for 24 hours before we placed them into incubators at 4°C, 12°C, or 26°C. We recorded worker survival weekly for six weeks by counting and removing dead individuals in each unit. We analyzed treatment effects on ant survivorship with a Wilcoxon 2-sample Test.

### Coexistence of *L. humile* and *P. chinensis* across *L. humile's* invaded range

We investigated the potential for coexistence of *L. humile* and *P. chinensis,* as well as the potential replacement of *L. humile* by *P. chinensis* across *L. humile-*invaded habitats. We sampled 14 locations (11 in North Carolina and 3 in South Carolina) where *L. humile* were documented in the last 10 years to understand how likely *L. humile* persist in its invaded range in the absence of *P. chinensis*. Historical records were obtained from the Departments of Entomology at North Carolina State University and Clemson University, SC. We re-sampled each location by placing bait cards (7.6×12.7 cm) every 5 m along a 25 m transect and recorded species presence and abundance every 15 minutes for 2 hours. Bait consisted of 0.5 g equal parts tuna/honey mixture. We also removed leaf litter (0.5 m^2^) from four points across each study location for Winkler extraction and recorded ant species presence and abundance in leaf litter.

## Results

### Evidence of *L. humile* and *P. chinensis* across seasons

In every year, more clusters of *P. chinensis* workers were recorded in March–April compared with each previous month (2009: F_1,10_ = 74.12, p<0.01; 2010: F_1,10_ = 42.19, p<0.01; 2011: F_1,10_ = 26.70, p<0.04). More clusters of *L. humile* were recorded in May–June compared to the previous months April–May (May–June 2009, F_1,10_ = 17.84, p<0.01; May–June 2010, F_1,10_ = 14.61, p<0.01; May–June 2011, F_1,10_ = 16.92, p<0.01). *Pachycondyla chinensis*, therefore, is present in the field two months prior to the period where *L. humile* populations increase significantly (Figure 1).

**Figure 2:**
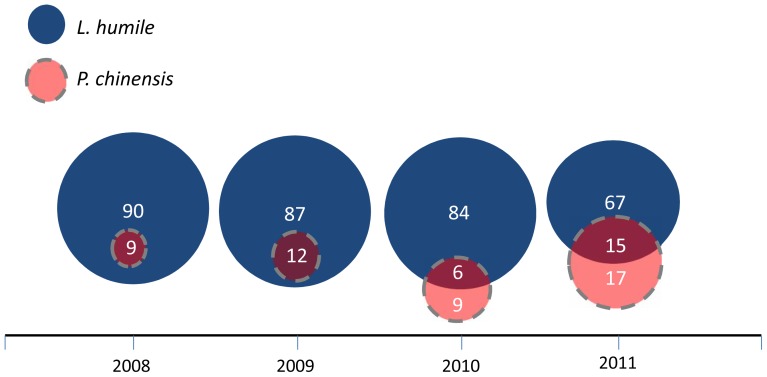
Venn diagrams representing the number of locations with *L. humile* (blue) and *P. chinensis* (pink with dashed border), in 2008, 2009, 2010, and 2011. Overlapping circle areas represent tree bases with *P. chinensis/L.humile* species overlap. Numbers within circles depict the number of trees with each status (*P. chinensis-*only, *L. humile-*only, or *P. chinensis/L. humile* overlap) for each measurement period. In 2008 and 2009, there were no *P. chinensis-*only locations (inner circles depict species overlap locations).

**Figure 3:**
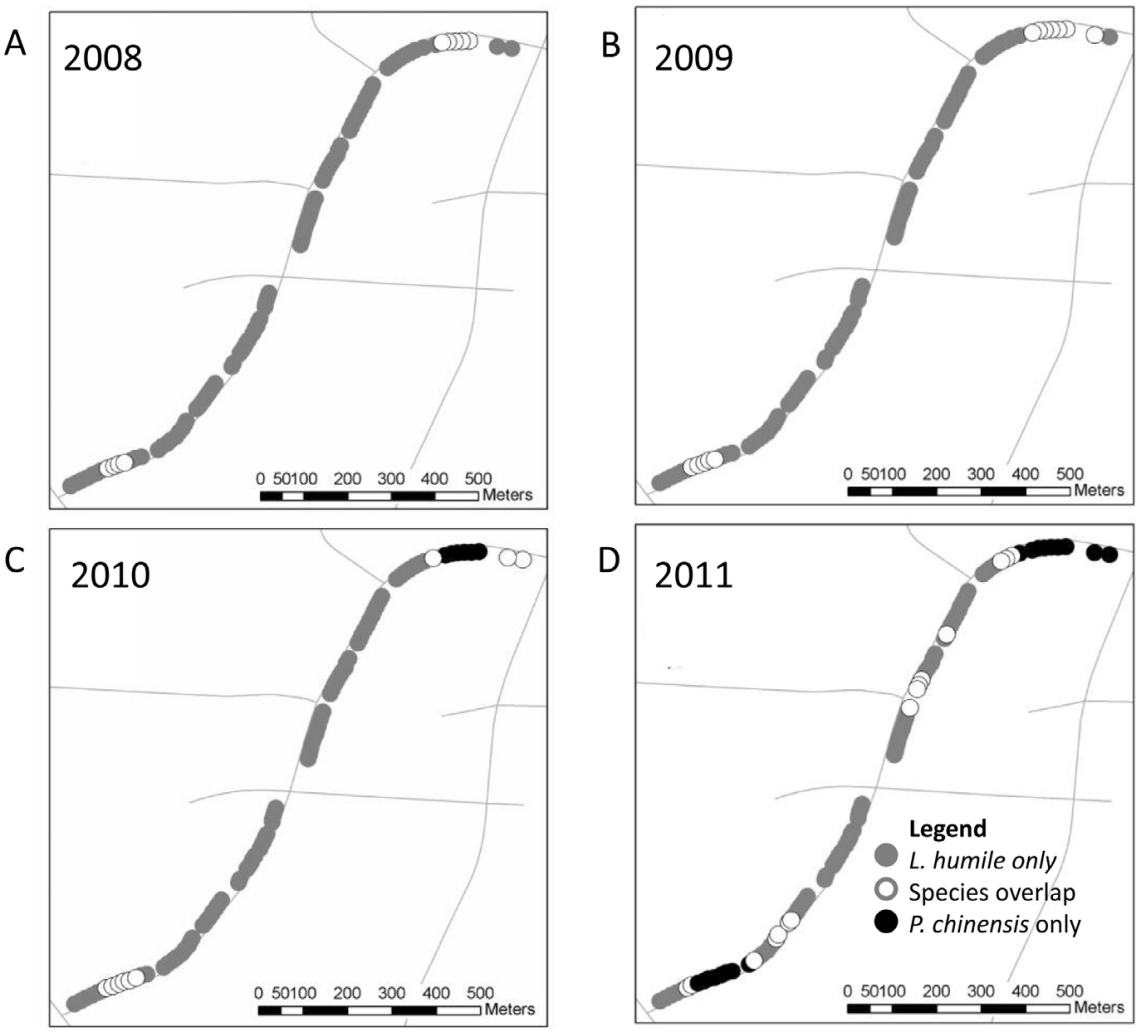
Records of *L. humile-*only (gray), *P. chinensis/L. humile* overlap (open) and *P. chinensis-*only (black), at the bases of trees across an invaded office park over four years: A) 2008, B) 2009, C) 2010 and D) 2011.

Our four-year census (2008–2011) at the bases of trees (n = 99) at our Morrisville, North Carolina field site revealed that the number of locations with *L. humile-*only (“3”) decreased significantly (from 90 locations to 67 locations) over four years while the number of locations with *P. chinensis-*only (“1”) increased significantly, and the number of *P. chinensis/L. humile* overlapping locations (“2”) fluctuated across years (from 0 tree bases in year one to 17 tree bases in year four and from 9 tree bases in year one to 15 tree bases in year four, respectively; χ^2^_1df_ = 248.39, p<0.01; Figures 2,3). *Pachycondyla chinensis* appeared at more locations in year four (2011) than all prior years, an increase of 17% over year three (Figures 2, 3). *Pachycondyla chinensis* spread was somewhat continuous during the first three years (Figure 3A–C). At year four we detected *P. chinensis* at discontinuous sites (Figure 3D), suggesting short-distance jump dispersal. However, since we did not sample habitat adjacent to our focal sites, *P. chinensis* may have emigrated from bordering areas rather than from distant sampling sites.

### Behavior and competition at baits

**Figure 4:**
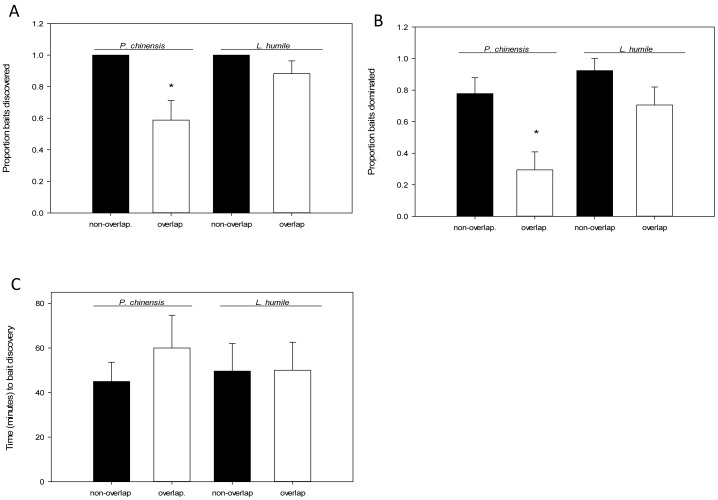
The mean proportion (±SE) of baits discovered (A), mean proportion (±SE) of baits dominated (B) and time (mean ± SE minutes) to bait discovery (C) by *P. chinensis* and *L. humile* in areas of species overlap or non-overlap (*p<0.05, Tukey HSD ANOVA, within species comparison).

*Pachycondyla chinensis* discovered (F_3,61_ = 6.47, p = 0.01; Figure 4A) and dominated (F_3,61_ = 6.34, p = 0.01; Figure 4B) fewer baits than *L. humile* in locations of species overlap and discovered fewer baits than in locations solely occupied by *P. chinensis* (Figure 4 A–B). However, there was no difference in time to bait discovery between either species, regardless of whether or not they were from areas of species overlap (f_3,52_ = 0.25, p = 0.86; Figure 4C). We observed no aggressive behaviors among ants at baits.

### Effect of colony size and order of establishment on ant species displacement and worker survival

Propagule pressure and resident status each contributed to the success of *P. chinensis* in occupying optimal nest space (Table 1). *Pachycondyla chinensis* displaced significantly more *L. humile* at higher *P. chinensis: L. humile* ratios and when *P. chinensis* established nests first (for all “displaced”: F_5,54_ = 37.97, p<0.01). In treatments with no *P. chinensis*, *L. humile* remained in their primary nest for the duration of the study and no workers or queens died.

**Table 1:** Nest defense and survival of *L. humile* at various *P. chinensis ∶ L. humile* ratios with *L. humile* as resident or intruder.

*P. chinensis* ∶ *L. humile* ratio	*L. humile* intruder		*L. humile* resident	
	Displaced	Surviving	Displaced	Surviving
**1∶1**	1 A	0±0 C	0.7±0.15 AB	0.45±0.13 BC
**1∶2**	0.9±0.1 A	0.18±0.06 CB	0.4±0.16 BC	0.86±0.07 A
**1∶5**	0.8±0.13 AB	0.49±0.1 A	0 C	1±0 A

Data expressed as proportion of total replicates (±SE) (n = 12). Significant differences (displaced or surviving) within treatment columns (resident or intruder) are denoted by letters (Tukey-Kramer comparison of means, p<0.05).

*Pachycondyla chinensis* killed more *L. humile* workers at equal densities or when *P. chinensis* occupied nests first (for all surviving: F_5,54_ = 21.38, p<0.01). *Pachycondyla chinensis* killed fewer *L. humile* workers when *L. humile* secured nests first and when *P. chinensis* ∶ *L. humile* ratios were greater than 1∶1 (Table 1).

In treatments where *L. humile* were the first to become established, *L. humile* killed most *P. chinensis* workers at a 1∶5 *P. chinensis* ∶ *L. humile* worker ratio (t_3,36_ = 17.12, p<0.01). However, when *P. chinensis* established nests first, few *P. chinensis* were killed, even when greatly outnumbered by *L. humile* (t_3,36_ = 16.35, p<0.01; Table 2).

**Table 2:** Survival of *P chinensis* at various *P. chinensis ∶ L. humile* ratios when *L. humile* is either the resident or intruder.

*P. chinensis* ∶ *L. humile* ratio	*L. humile* resident	*L. humile* intruder
**1∶1**	0.99±0.01 A	0.99±0.01 A
**1∶2**	0.96±0.14 A	0.87±0.05 B
**1∶5**	0.11±0.08 C	0.82±0.1 B

Data expressed as mean proportion (±SE). Significant differences within treatment columns denoted by different letters (Tukey-Kramer comparison of means, p<0.05).

### Reappearance of *L. humile* following removal of *P. chinensis* from the field

**Figure 5:**
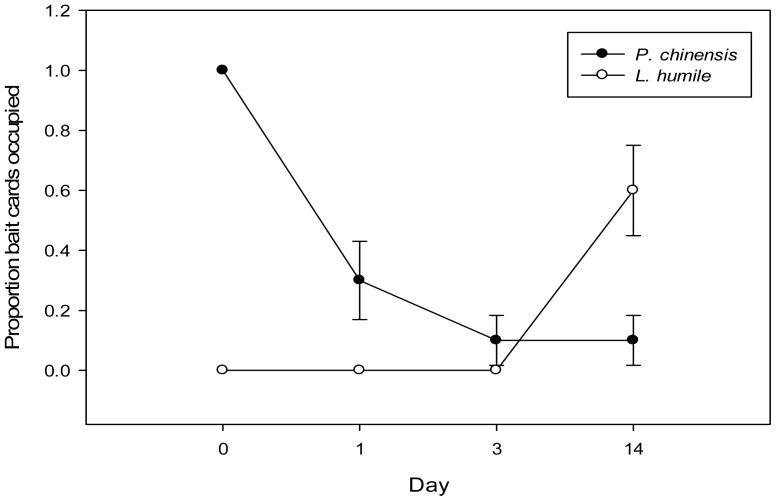
Mean (±SE) proportion of baits occupied by either *P. chinensis* (○) or *L. humile* (•) in plots chemically-treated to remove *P. chinensis* 0, 1, 3, and 14 days after treatment (χ^2^_1_ = 9.82, p = 0.01).

No *L. humile* were recorded at bait cards (n = 28) in *P. chinensis*-occupied plots. We also found no *L. humile* at baits (n = 16) in plots that did not receive insecticide treatment for the entire study. *Pachycondyla chinensis* were recorded at all (16/16) baits in untreated plots for the duration of the study, but at zero baits one day after insecticide treatment and at only 25% (3/12) of baits in treated plots 14 days after insecticide treatment. Furthermore, 14 days post-treatment, 50% of the baits in the treated area previously occupied by *P. chinensis* were now visited by *L. humile* compared to untreated plots, which remained free of *L. humile* (χ^2^_1_ = 9.82, p = 0.01; Figure 5). After controlling for treatment, there was no significant association between site and *L. humile* presence (p = 0.27).

### Cold tolerance of *L. humile* and *P. chinensis*

**Figure 6:**
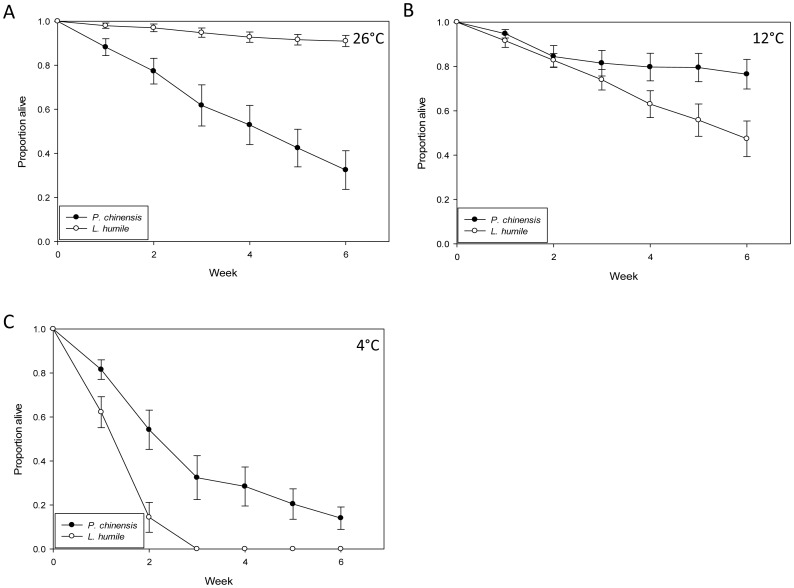
The proportion (mean ±SE) of *P. chinensis* and *L. humile* workers surviving at A) 26°C B) 12°C and C) 4°C.

More *L. humile* survived at 26°C than *P. chinensis* (Wilcoxon 2-sample Test: χ ^2^ = 47.36; n = 10; p<0.01); Fig. 6A). In contrast, *P. chinensis* survival exceeded that of *L. humile* at 12°C (Wilcoxon 2-sample Test: χ ^2^ = 26.5; n = 10; p<0.01; Fig. 6B) and at 4°C (Wilcoxon 2-sample Test: χ ^2^ = 47.87; n = 10; p<0.01; Fig. 6C), with all *L. humile* workers dying by week three at 4°C. We suggest that *P. chinensis* may be more cold-tolerant than *L. humile*.

### Coexistence of *L. humile* and *P. chinensis* across *L. humile's* invaded range

Our bait card and leaf litter surveys of 14 re-sampled *L. humile* sites revealed *L. humile* at seven and *P. chinensis* at two of those sites. *Pachycondyla chinensis* was also recovered from one re-sampled site in which *L. humile* was absent. Thus, *P. chinensis* and *L. humile* overlap in portions of their-invaded range.

## Discussion

We provide the first evidence for small-scale displacement of an established widespread invasive ant by a more recently introduced exotic ant species. *Pachycondyla chinensis* was first observed in 2008 at the edge of an established (>12 yr-old) *L. humile* population and has steadily expanded into habitat previously occupied by *L. humile*. The return of *L. humile* to areas where *P. chinensis* was deliberately eliminated strongly suggests that *P. chinensis*—and not another factor such as unsuitable habitat—was responsible for the decline of *L. humile*. While propagule pressure may aid in the establishment of an invasive species, we suggest that priority in nest establishment is also a predictor of habitat dominance. We suggest that by possessing a broader seasonal activity envelope and surviving at lower temperatures than *L. humile*, *P. chinensis* establishes earlier in the season before *L. humile* colonies grow and expand.

As exotic organisms invade new habitat, they often encounter and interact with established invaders [48], [74]. However, evidence of a newly introduced species displacing an established invader is rare [75]. Most studies on ant invader-invader interactions focus on short-term competitive interactions [76]–[78], predicting long-term effects of these interactions [79]. Here, we combined laboratory studies with extended field surveys to reveal the consequences of, and possible mechanisms underlying, invasion succession.

A major consequence of invasion is the cascading impact the invading organism has on its environment. Negative influences on native taxa from a primary invasion can promote entry of a secondary invader [48], [80]. For example, land snails (*Achatina fulica*) are more likely to invade areas inhabited by the invasive ant *Anoplolepis gracilipes*, which eliminates snail-eating land crabs [81]. The displacement of potential *P. chinensis* competitors by *L. humile* through phylogenetic pruning by competitively excluding native ant taxa may be facilitating *P. chinensis* establishment [46], [82], [83]. However, this scenario does not explain establishment of *P. chinensis* in natural environments harboring few, if any, exotic ants [39]. Therefore, while *L. humile* may provide an entryway to *P. chinensis* invasion in some disturbed environments, other factors contribute to invasion success.

A more likely explanation for the successful establishment and spread of *P. chinensis* in central North Carolina relates to its activity throughout much of the year particularly in early spring when *L. humile* workers are largely inactive. This study adds to a growing body of knowledge highlighting the importance of climatic suitability for invasion success, but it is unique by underscoring the importance of climatic suitability in resisting secondary invasion [44], [45], [47], [84]–[87]. Seasonal effects on distribution and range expansion could facilitate the return of certain native ant species during periods when *L. humile* is absent [44]–[47]. We suggest that *L. humile* early-season scarcity, coupled with the broader temperature tolerance of *P. chinensis* with its appearance during March and April, promotes the establishment and spread of *P. chinensis*. In our cold tolerance experiments we included *L. humile*, but not *P. chinensis* queens. While *L. humile* queens require workers for survival [73], we do not know if *P. chinensis* queens can establish nests without workers. If claustral founding does occur in *P. chinensis* and these queens are more cold-tolerant than workers we would expect even greater differences in nest survival at low temperatures between the two ant species.

Propagule pressure is often touted as a driving force behind the successful establishment of introduced species [15], [16], [59], [88], [89] and propagule pressure and nest establishment priority can facilitate successful *P. chinensis* establishment, with declining *L. humile* colony size in winter [90]. Yet, propagule pressure is not necessarily a good predictor of invasion success, particularly in ant species displaying behavioral plasticity [91]. We provide laboratory evidence that propagule size plays a role in the establishment of *P. chinensis* within *L. humile* territory, yet nest establishment priority appears to be a better predictor of *P. chinensis* success.

Here, we elucidate factors driving the establishment of *P. chinensis*, as well as document the spread of *P. chinensis*. Still, the mechanisms underlying the persistence of this behaviorally subordinate species in a landscape inundated with a behaviorally and numerically dominant ant remain unclear. We show that in areas of coexistence, *L. humile* dominates more food resources and displaces *P. chinensis* present at those resources. Behavioral and numeric dominance are corollaries of an invasive organisms' ecological success [9], [92]. While dolichoderine ants like *L.humile* include many invasive species, comparatively few ponerines are invasive [93]. Furthermore, native non-hypogaeic ponerine species are generally eliminated by invasive ants [35], [94], due in part to their relatively small colonies [95], [96], generally solitary foraging behavior [97]–[99], and characteristic subdominant or subordinate behavior [100]. Subordinate behavior in particular can facilitate coexistence between invasive and native ant species, and this could explain, in part, habitat overlap between *P. chinensis* and *L. humile* [101], [102]. In our experiments, we observed that *P. chinensis* use both subordinate behaviors (running away) and aggressive behaviors (stinging) when in interacting with *L. humile*. Our ongoing research addresses the context dependency and consequences of these behaviors. Ponerine ants are particularly amenable to coexistence, with many species permitting cohabiting myrmecophiles or sharing nest space or trail markings with other ant species [103]–[105]. Our field surveys revealed that *P. chinensis* populations overlap with *L. humile* before *L. humile* is ultimately displaced. Understanding the mechanisms promoting the initial overlap, or coexistence, between these two species will help explain how *P. chinensis* populations are sustained as *L. humile* numbers increase in summer months.

In diverse community mosaics, many ant species are able to coexist through resource partitioning. Whereas some coexisting ant species display different diel and/or seasonal activity patterns [106], [107], others coexist by partitioning resources such as habitat space [108] or food [109]. Here, however, both *L. humile* and *P. chinensis* have nest requirements and forage actively during most months. Because *L. humile* actively forage 24 hours a day [36], [110], they also have a presumed overlap in diel activity patterns with *P. chinensis* during seasonal periods of high activity. Our preliminary data suggest that, while *P. chinensis* are found outside the nest all day, they are most actively foraging between 0800 and 1200 h (ESR unpublished data). *Pachycondyla chinensis* is both a predator and scavenger [111]. *Linepithema humile* consumes prey as well, particularly during peak colony growth [112]. It therefore seems unlikely that resource partitioning drives coexistence between these two species.

Invasive ants are generally not observed until their negative ecological impacts on native taxa are recognized. Here, we follow the spread of an incipient *P. chinensis* population as it displaces a portion of a colony of *L. humile* within a few years. We suggest that this occurs in part because *P. chinensis* is active when *L. humile* is dormant. *Pachycondyla chinensis* may similarly gain a foothold in natural undisturbed habitats by displacing native ants before a defendable colony size is attained. By recognizing the instrumental influences in an invasion dynamic, we can attempt to mitigate current and forestall future negative environmental impacts caused by invasive species.
